# CACPU-Net: Channel attention U-net constrained by point features for crop type mapping

**DOI:** 10.3389/fpls.2022.1030595

**Published:** 2023-01-04

**Authors:** Yuan Bian, LinHui Li, WeiPeng Jing

**Affiliations:** The College of Information and Computer Engineering, Northeast Forestry University, Harbin, China

**Keywords:** artificial intelligence, smart agriculture, crop type mapping, remote sensing, semantic segmentation, attention mechanisms

## Abstract

Crop type mapping is an indispensable topic in the agricultural field and plays an important role in agricultural intelligence. In crop type mapping, most studies focus on time series models. However, in our experimental area, the images of the crop harvest stage can be obtained from single temporal remote sensing images. Only using single temporal data for crop type mapping can reduce the difficulty of dataset production. In addition, the model of single temporal crop type mapping can also extract the spatial features of crops more effectively. In this work, we linked crop type mapping with 2D semantic segmentation and designed CACPU-Net based on single-source and single-temporal autumn Sentinel-2 satellite images. First, we used a shallow convolutional neural network, U-Net, and introduced channel attention mechanism to improve the model’s ability to extract spectral features. Second, we presented the Dice to compute loss together with cross-entropy to mitigate the effects of crop class imbalance. In addition, we designed the CP module to additionally focus on hard-to-classify pixels. Our experiment was conducted on BeiDaHuang YouYi of Heilongjiang Province, which mainly grows rice, corn, soybean, and other economic crops. On the dataset we collected, through the 10-fold cross-validation experiment under the 8:1:1 dataset splitting scheme, our method achieved 93.74% overall accuracy, higher than state-of-the-art models. Compared with the previous model, our improved model has higher classification accuracy on the parcel boundary. This study provides an effective end-to-end method and a new research idea for crop type mapping. The code and the trained model are available on https://github.com/mooneed/CACPU-Net.

## 1 Introduction

Since the advent of satellite remote sensing, land cover classification has been an essential and active topic in land-use science and agriculture Ren et al. (2022). Typically, the land cover classification includes cropland class but lacks fine-grained classification of crop types Xiong et al. (2017). In agricultural applications, crop type classification is of great importance for crop yield prediction and agricultural disaster estimation. Accurate crop type mapping is a necessary prerequisite for developing many smart agricultural technologies, as well as a technical means for agricultural policy-making and sustainable development.

With the rapid development of Earth Observation Satellites, the resolution of remote sensing data has been continuously increasing, which provides an opportunity for high-resolution and high-precision crop type mapping. Recently, more and more research on crop type mapping has emerged. However, crop type mapping is challenging due to crop diversity, inter-class spectral similarity, intra-class variability, and other factors (Zhong et al., 2014).

In the traditional methods of crop type mapping, machine learning methods, such as random forest algorithm, have always been the mainstream (Yang et al., 2019; Pott et al., 2021). With the rapid development of deep learning, some deep learning research has also begun to emerge in crop type mapping and has demonstrated its advantages over machine learning. Using Landsat Enhanced Vegetation Index (EVI) time series as data, Zhong et al. (2019) replaced the traditional machine learning method with a simple Fully-connected Neural Network (FNN) and achieved better performance. This proves that deep learning is superior to machine learning in crop type mapping. However, the method they proposed still requires manual intervention. The input data of FNN are the features that need to be extracted through complex manual preprocessing, and it is highly dependent on the prior knowledge of the professional field.

To overcome the limitation of manual feature extraction and due to deep learning has shown significant advantages over other methods in feature extraction in various fields, the other part of the studies on crop type mapping based on deep learning automatically extracts features from remote sensing images by end-to-end Convolutional Neural Networks. Influenced by the habit of data selection in traditional crop type mapping methods (Cai et al., 2018), these studies still use time series data to generate datasets, although sometimes this is unnecessary. This way of data selection makes researchers mainly focus on the temporal features of time series data and have designed a series of time series models. Rustowicz et al. (2019) used 2D U-Net+CLSTM and 3D U-Net to map crop types for smallholder farms in Africa, but it mainly improves the performance of the model by integrating other data sources and pays insufficient attention to the classification accuracy of land parcel boundaries. Garnot et al. (2022) proposed U-TAE, which combines 3D U-Net with a time attention module called TAE to enhance the ability to extract temporal features. The research of Garnot and Landrieu (2021) showed that deep learning models designed for time-series data perform poorly on single-temporal satellite image data in their ablation experiments. However, the difficulty of making a multi-temporal dataset is much higher than that of a single-temporal dataset, which undoubtedly hinders the implementation of agricultural applications. Deep learning has a good extraction effect for spectral features and spatial features and has achieved favorable results in pixel-level classification without using temporal features. We note the success of deep learning on 2D semantic segmentation targeting large public datasets of typical color images, land cover datasets, and medical images. In the general computer vision community, we associate the crop type mapping of single-source single-temporal remote sensing data with 2D semantic segmentation to improve our task based on its research results.

Some of the earliest works applying deep learning to semantic segmentation started with the Fully Convolutional Network (FCN) proposed by Shelhamer et al. (2017), which used convolutional neural networks as the basic architecture to perform supervised classification of pixels in raster images, and achieved remarkable results. Ronneberger et al. (2015) presented a U-Net, designed for medical image segmentation, and it is the first semantic segmentation network trained on a small dataset. Badrinarayanan et al. (2017) proposed SegNet, which was the first to propose the idea of encoding and decoding, and its encoder-decoder structure has been used in semantic segmentation until now. Chen et al. (2018) proposed DeepLab series of network models, used atrous convolution and introduced atrous pooling, used ResNet He et al. (2016) as the backbone, and used Xception Everingham et al. (2014) for the segmentation task, which achieved the state-of-the-art performance on the publicly available dataset VOC2012 Everingham et al. (2014) at that time.

Following the convolutional neural network, the attention mechanism has gained extensive attention. The attention mechanism can combine with the convolutional neural network well. The mainstream attention mechanisms include channel attention, spatial attention, temporal attention, and branching attention, all with great success. In medical image segmentation, the attention mechanism often combines with the model to handle class imbalance. Guo et al. (2021) proposed SA-UNet on Real Vessel Segmentation, which added a spatial attention module between the encoder and decoder, effectively improving the model’s ability to classify blood vessels and backgrounds. Yeung et al. (2021) proposed Focus U-Net and designed an attention module called Focus Gate, which can encourage learning of salient regions and suppress learning of irrelevant background regions. In land cover, Li et al. (2022) proposed a MAResU-Net that introduced a multi-stage CAM attention module and achieved state-of-the-art performance on the VAIHINGEN dataset ISPRS (2018). Inspired by the above studies, we tried a variety of different attention mechanisms in our method to find a more suitable attention module for crop type mapping to improve the model performance. In recent times, a type of special spatial attention called transformer self-attention has appeared in the researchers’ view. Almost all the current state-of-the-art semantic segmentation networks use this transformer structure. However, it is frustrating that the transformer does not get good results in training with a small dataset (Dosovitskiy et al., 2021), making it difficult to adapt to crop type mapping.

Our study selected Sentinel-2 satellite imagery as the data source. Sentinel-2 satellites are polar-orbiting multi-spectral high-resolution imaging satellites used for land monitoring to provide imagery such as vegetation, soil and water cover, inland waterways, and coastal areas. The satellites are divided into Sentinel-2A and Sentinel-2B, respectively, launched on June 23, 2015, and March 7, 2017. Sentinel-1 and Sentinel-2 satellites offer near-real-time images with high spatial (10–60 m) and temporal (1–5 days) resolution (Pott et al., 2021). The study of Ren et al. (2022) demonstrates the advantages of Sentinel-2 satellite in crop type mapping. Single-source single-temporal Sentinel-2 satellite crop type mapping dataset has low difficulty to produce, good effect, no manual intervention, and better meets the needs of agricultural automated monitoring.

In this paper, we designed CACPU-Net, which is a two-way end-to-end crop type mapping network with an encoder-decoder structure based on point features and spectral features. On the single-source single-temporal Sentinel-2 satellite imagery dataset, it is significantly better than other deep learning methods, reaching 93.74% overall accuracy. This proves that the network we designed can meet the application requirements, and also shows that deep learning has great potential for future applications of crop type mapping. The main contributions of this paper are as follows:

- We fully apply the mainstream 2D semantic segmentation models to multi-crop type mapping and improved the U-Net, which has the best performance, into a two-way network, further improving the performance of the model in crop type mapping, especially in the boundary of the parcel. And we produce a single-Source and single-temporal Autumn Sentinel-2 satellite crop type mapping dataset.
- We evaluated our scheme and defined CACPU-Net, a new state-of-the-art method to crop type mapping.
- We verified whether many proposed modules, such as mainstream attention modules, different loss functions, and so on, are effective in crop type mapping.
- We show that single-temporal remote sensing images of the harvest period can be effectively applied to crop type mapping.

## 2 Materials and methods

### 2.1 Data collection

All data used in our experiments were collected at the BeiDaHuang YouYi in Heilongjiang Province, China, with geographic coordinates ranging from 46°28 ‘15 “to 46°58’ 39” N and 137°27 ‘50 “to 132°15’ 38” E.

**Figure 1:**
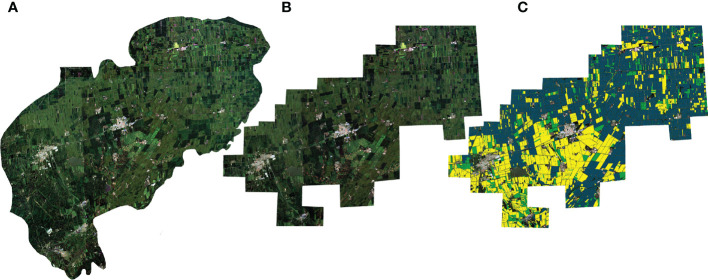
**(A)** is the original full-frame image, **(B)** is the pre-processed full-frame image, and **(C)** is the annotated pre-processed full-frame image.

The data source is the 10m high-resolution remote sensing image of the Sentinel-2 satellite, and the collection date is August 17, 2021. The label data is collected by the local insurance company. In the experiments, we used four bands of red, green, blue, and near-infrared light from satellite images. The size of the original remote sensing image is 5505×4280, as shown in **Figure 1**.

### 2.2 Dataset

**Figure 2:**
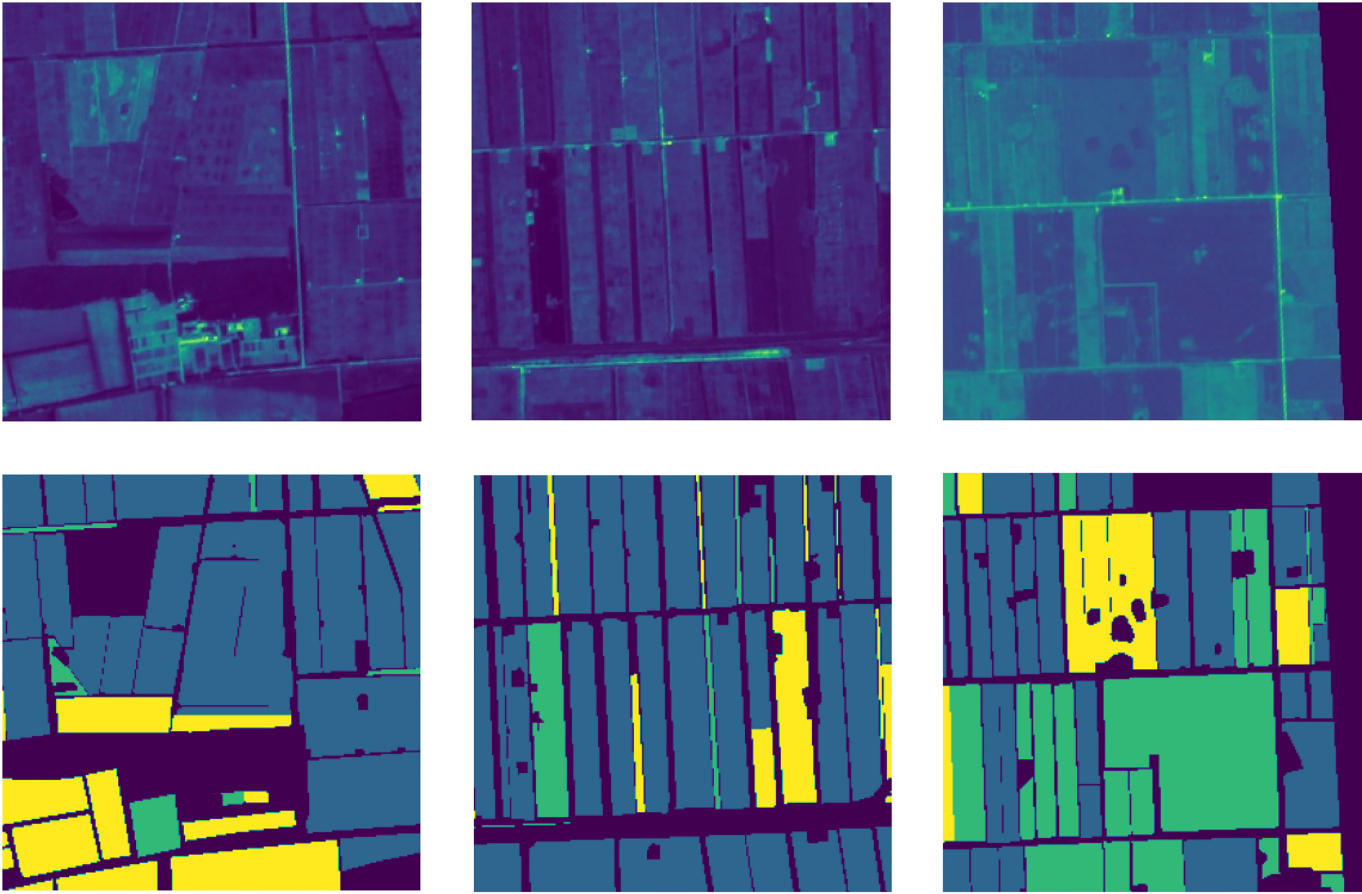
The upper part is the image patches, and the lower part is the corresponding ground truth.

Some uninsured farmland has no labels of crop type in the corresponding pixels of the image. So we manually added the rectangular mask on some image areas for the original image to reduce the impact of the areas lacking labels on model performance. The masked image is shown in **Figure 1B**. To better identify the main crops, we removed the peanut and wheat categories that could not be recognized by the model due to the small number of pixels and retained the rice, Maize, soybean, and non-farmland categories. We cut the original image into 256×256 size image patches in the sliding window manner with a stride of 256 to avoid cross-contamination between training, validation, and testing datasets. For the cropped patches, we discard the patches in which the proportion of masked pixels is higher than 15%. Some examples of the image patches produced by the above processing are shown in **Figure 2**.

Our final dataset has 143 training image patches, 17 validation image patches, and 17 testing image patches. The split ratio of the dataset is approximately 8:1:1. All our experiments are performed with 10-fold cross-validation. And in each experiment, the dataset is randomly assigned according to the above split ratio. The specific number of pixels is shown in **Table 1**.

**Table 1:** Dataset statistics.

Type	Rice	Maize	Soybean	Other	Total
**Pixel Num**	4,685,724	2,833,795	925,493	15,116,388	23,561,400

Rice, Maize, and Soybean are the main crop type in the dataset we produced. The parcels of other crop types or the parcels without insurance are labeled in the Other type. In addition, the Other type also includes all non-farmland parcels, including towns, waters, wastelands, roads, etc.

### 2.3 CACPU-Net

**Figure 3:**
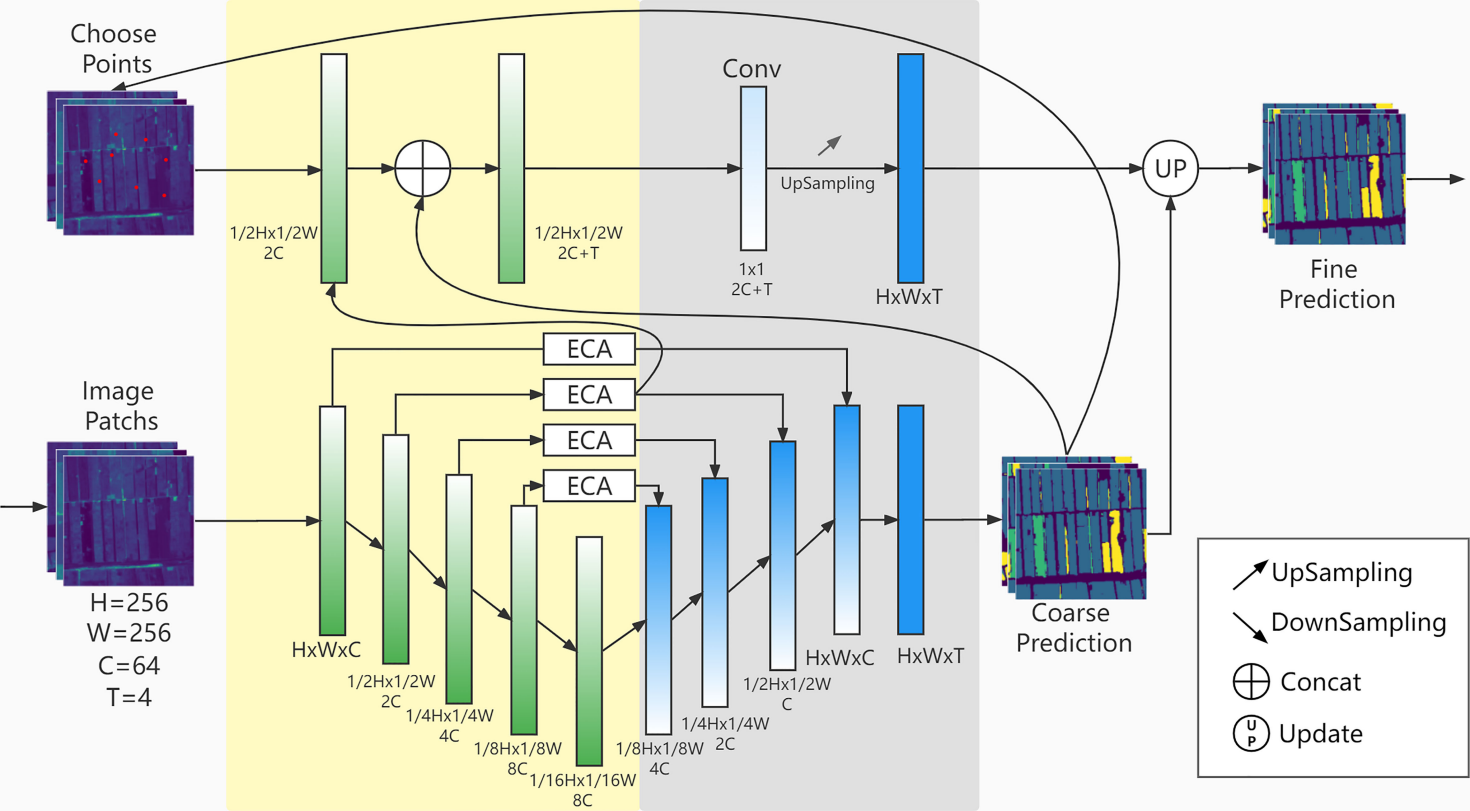
CACPU-Net. The yellow part is the network encoder, and the gray part is the network decoder. The upper part is the CP Module structure, and the lower part is the Baseline Network structure.

CACPU-Net follows the encoder-decoder structure (**Figure 3**), a design idea obtained from semantic segmentation networks. The input image is encoded using the 5-layer encoder of U-Net (Ronneberger et al., 2015) combined with the Efficient Channel Attention (ECA) module (Wang et al., 2020). To improve the activation degree of pixels, we use the PReLU (He et al., 2015) activation function in the encoder to replace the original ReLU activation function. The decoder part of the network has two branches, the master branch is a 4-level cascaded bilinear interpolation upsampling module. The second branch, which is named Constrain of Point (CP) Module, extracts one-dimensional point features from the second-layer encoder, and inputs them into a separate MLP module to obtain the final result.

#### 2.3.1 Baseline network encoder

This module is mainly composed of a five-layer structure. The first layer is a double-layer convolution module that converts the 4-band original image into a 64-channel feature map and keeps the image size unchanged. Each subsequent layer is a down-sampling module, which reduces the resolution of the input image to 1/2 and expands the channel to 2 times the original. The double-layer convolution module consists of two 3×3 convolutional layers, activation functions, and BN layers connected. The downsampling module consists of a max-pooling layer in series with the double-layer convolution module. The encoder formula is as follows:


(1) $$X_{en}^{i}={\left[sign\left(BN\left(C(X^{i})\right)\right)\right]}^{2},i=1,\ldots ,5,$$

where *X^i^*/ $X_{en}^{i}$ is the input/output of the *i*th encoder, *C*(·) stands for the convolution operation, *BN*(·) for the Batch Normalization operation, *sign*(·) for the activation function, and [·]^2^ represents the above calculation of the double layer structure. The formula we use for the activation function PReLU is shown below:


(2) $$sign(x)=\{\begin{array}{ll} x, & x>0\\ \alpha x, & x\leq 0 \end{array}$$

#### 2.3.2 Baseline network decoder

The baseline network decoder is nearly symmetrical in structure with the encoder and is 4 up-sampling modules. The upsampling module is a bilinear upsampling layer concatenated with the double-layer convolutional module. The input of each level of the upsampling module is the feature map of the current resolution and the feature map of the previous level of resolution. After sampling the current resolution feature map to increase the resolution, the feature map of the previous resolution is fused with the feature map of the previous level through the concatenate operation, and then the number of channels is reduced to 1/4 through the double-layer convolution module. The final feature map is 64 channels, and the final pixel-level classification results are obtained through a 1×1 convolutional layer.

#### 2.3.3 ECA module

**Figure 4:**
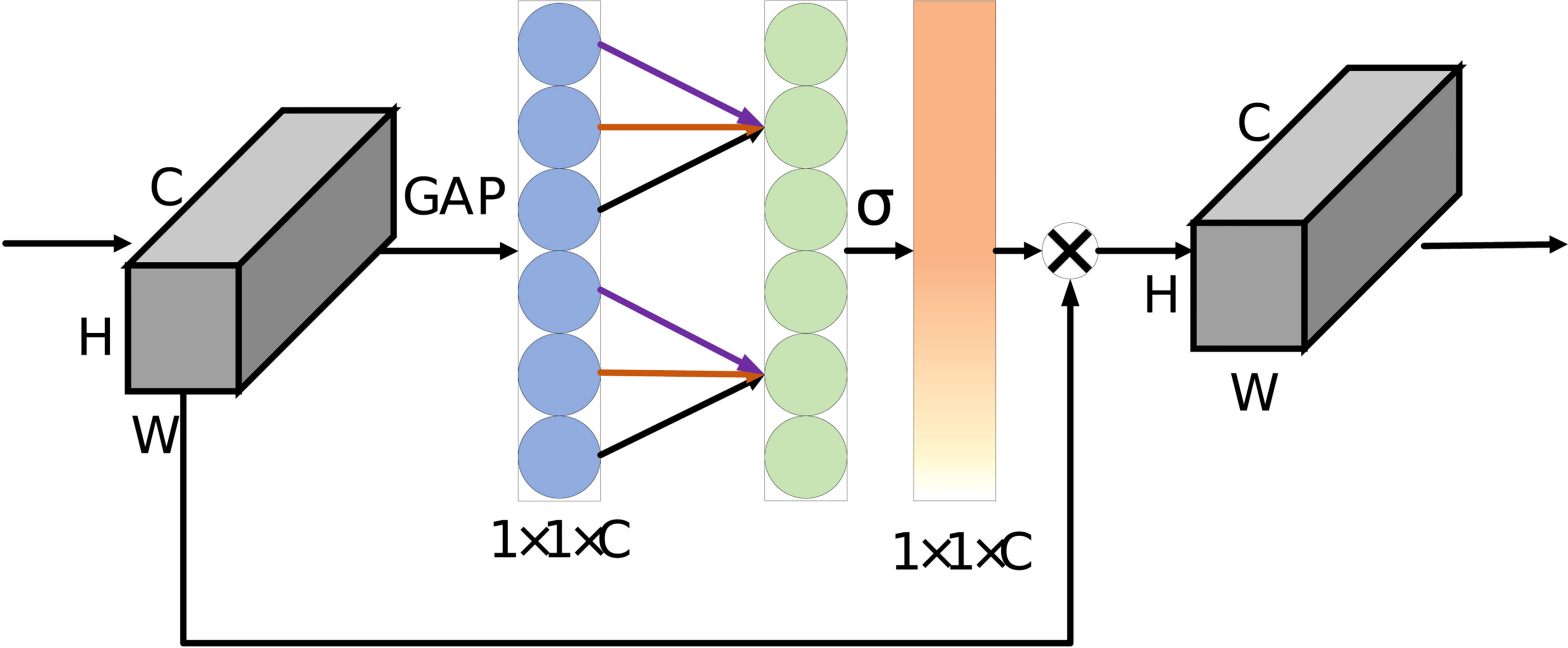
The ECA module takes the C×H×W feature map as input, compresses features through global average pooling (GAP), and uses 1×1×C 1-dimensional convolution with kernel size of 3 to obtain the channels’ attention weight. Finally, the attention map is obtained by Dot between the attention weight obtained and the original input of the module. σ is Sigmoid function.

The Attention module of our network uses the ECA module (as shown in **Figure 4**). ECA is a type of channel attention. Channel attention enables the model to select the channel of interest by adding a learnable weight to each channel of the feature maps. This can improve the impact of key features on the prediction results, suppress the impact of irrelevant features or noise, and thus improve the accuracy of crop type identification.

ECA’s formula is as follows:


(3) $$\omega^{i}=\sigma (C1D_{3}(x^{\prime i})),\omega^{i}\in {\text{Ω}}^{4}$$

where the Ω^4^ represents the attention modules corresponding to four different resolutions, *x*′ is the feature map after global average pooling, *ω* is the weight of all channels, *σ* is the Sigmoid function, *C*1*D* is the 1-dimensional convolution kernel, and its subscript 3 is the kernel size of the convolution kernel.

Finally, *ω* and the original feature map *x* generate the final attention map *A* by dot operation. The formula is as follows:


(4) $$A^{i}=\omega^{i}•x^{i},A^{i}\in {\text{Ω}}^{4}$$

At the same time, we use multi-stage attention. The feature maps at the first four levels of resolution all use attention modules, and the number of channels in the original feature map is not changed. The detailed embedding of attention modules in the network structure can be seen in **Figure 3**.

#### 2.3.4 CP module

The design inspiration for the CP module comes from PointRend (Kirillov et al., 2020). In our model, the CP module is the decoder module of the second branch. Its input is the feature array of the difficult-to-classify point in the image, and its output is the crop type corresponding to that point. The CP module has a separate loss function and a different network structure from the master branch of the model, so it can independently predict the types of selected points that are difficult to classify and improve the prediction accuracy of the whole model.

First, the CP module calculates the classification difficulty of each pixel of the master branch prediction result and selects the top k points that are the most difficult to classify. This calculation is obtained by the difference in the scores of each point type. And in our model, the k value is selected as 8096, and this number accounts for about 12% of the pixels of each image patch. This choice is because if the k value is too small, it will not affect the prediction results of the model, and if the k value is too large, it will excessively interfere with the prediction results of the master branch. Secondly, it extracts the features of points that are difficult to classify in the medium-resolution feature map. To correspond to the position of the point on the image patch, the feature map is upsampled to the same size as the image patch. Finally, it takes the features of the difficult-to-classify points and the prediction results of the corresponding points of the master branches as the input of the MLP module obtains the final prediction results of the difficult-to-classify points through training and covers the results of the points corresponding to the master branches.

### 2.4 Network training

During training, the masked pixels are ignored. This is because there should be no masked regions during inference in practical applications. Since we used 4-band data for network training, all of the models were not pre-trained. During the training process, we use Adam optimizer, and the learning rate is 0.0003. A total of 150 epochs of training are performed, and the batch size is 16. Training is performed on Tesla v100 GPU, and the training is interrupted in advance if the model performance does not improve within 20 epochs.

#### 2.4.1 Loss function

The design inspiration for our loss function comes from the research of medical image segmentation loss function. Ma et al. (2021) has evaluated more than 20 different loss functions. From its experimental results, it can be seen that cross-entropy, Dice, and its variants can achieve stable and good performance. Yeung et al. (2022) proposed Unified Focal loss, a Dice and cross entropy-based loss, which achieves the most advanced performance in five different medical image segmentation public datasets. In medical image segmentation, the loss function is mainly designed to solve the class imbalance problem between foreground (polyps, blood vessels, or other objects) and background regions. It is usually a pixel-level secondary classification, while in crop type mapping, it is a multi-crop classification, which brings some challenges.

During training, the two decoder branches of the network are trained with separate loss functions. The loss function used by the master branch is composed of Dice (Milletari et al., 2016) and cross-entropy with different weights. The formula is as follows:


(5) $$L=\alpha L_{c}+\beta L_{d},$$

where *L_c_* is the cross-entropy loss function and its weight parameter is *a*, and *L_d_* is the Dice loss function and its weight parameter is *β*.


(6) $$L_{c}=\sum_{i=1}^{C}\sum_{j=1}^{X}y_{ij}\log\;x_{ij},$$

(7) $$L_{d}=1-\frac{\sum_{i=1}^{C}\sum_{j=1}^{X}x_{ij}\cap y_{ij}}{\sum_{i=1}^{C}X},$$

where C is the number of channels of the feature map, X is the number of pixels in each channel of the feature map, *x_ij_* is the predicted value of pixel *j* of channel *i*, and *y_ij_* is the true value of pixel *j* of channel *i*.

Among them, the introduction of the Dice is mainly to handle class imbalance. The effect of the Dice has been verified in medical image semantic segmentation. The detailed weight ratio of the master branch loss function is shown in **Table 2**. Different from the master branch, the CP module only uses cross-entropy as the loss function.

**Table 2:** Loss function experiments.

Loss Ratio		OA (%)	AA (%)	mIoU (%)
CE	Dice			
1	–	92.43	90.34	83.51
–	1	**93.38**	91.67	**85.52**
1	1	93.25	**91.82**	85.36
1	2	93.34	90.85	84.52
1	5	93.35	90.92	84.77
1	10	**93.38**	91.01	84.79
2	1	93.27	91.24	84.98
5	1	93.15	90.98	84.66
10	1	93.21	91.21	84.65

The weights of the different loss functions are integers, not percentages. Bold font indicates the highest performance.

## 3 Results

### 3.1 Contrast experiments

In all experiments in this paper, three evaluation indicators are used, namely, Overall accuracy (OA), Average accuracy (AA), and mIoU. Among them, OA is the main evaluation indicator of our task, AA is mainly used to observe the average accuracy of various categories, and mIoU can better evaluate the misclassification of the model. Our study uses U-Net Ronneberger et al. (2015), Deeplab v3+ Chen et al. (2018), HR-Net, HR-Net (+OCR) Wang et al. (2021), and MAResU-Net Li et al. (2022) to conduct contrast experiments with our method. Detailed experimental results are shown in **Table 3**. It is worth noting that all models in the experiment are without pre-trained, the main factor is that the 4-band data for

this task is different from the 3-band RGB data of ImageNet, a general dataset for model pre-training. As can be seen from **Table 3**, our method has state-of-the-art performance. This performance is even more pronounced on mIoU, which is a 2.48% improvement over the second model. For each competing method of the contrast experiment, we made a non-parametric Wilcoxon’s test between it and our method to ensure that our method is superior to the other competing methods.

**Table 3:** Contrast experiments with various mainstream state-of-the-art semantic segmentation models.

Method	OA (%)	AA (%)	mIoU (%)
DeepLab v3+	79.29	73.50	60.87
HRNet+OCR	86.82	81.47	71.11
HRNet	87.80	82.42	72.62
MAResU-Net	89.55	86.34	77.18
U-Net	92.43	90.34	83.51
Ours	**93.74**	**91.75**	**85.99**

Bold font indicates the highest performance. The p-values for paired non-parametric Wilcoxon’s testing for our method versus each competing method are less than 0.05.

**Figure 5:**
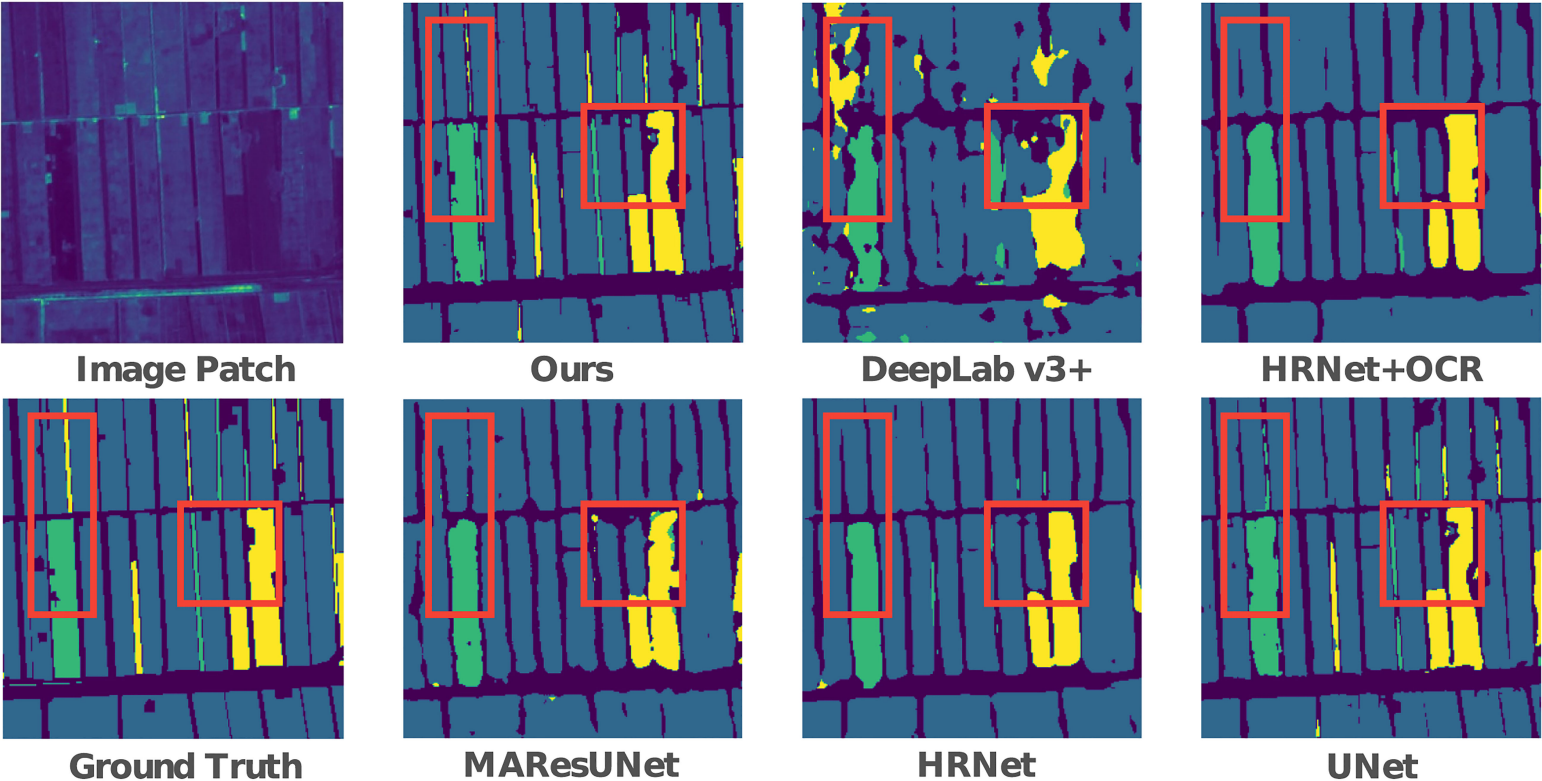
Visual prediction comparison of different methods.

The intuitive performance benefits of our method can be seen in **Figure 5**. It can be seen that all the other methods can complete crop type mapping to different degrees, except that Deeplab v3+ is unable to classify crops well. Compared with the inability of MAResU-Net, HR-Net, and HR-Net(+OCR) to classify long and narrow parcels, our method can be more accurate in this case. In addition, our method is more refined in the classification of the junction between farmland and non-farmland. Compared with U-Net, on the one hand, our method effectively reduces the misjudgment rate for the classification of long and narrow parcels. We can see that in the part circled by the red box, U-Net makes a misclassification of the entire parcel in the long and narrow parcel. On the other hand, our method performs better in the classification of irregular gaps at the edge of the parcels, which are also circled by the red box.

### 3.2 Ablation experiments

To analyze the influence of each module in the experiment on the final performance of the model, we performed ablation experiments on each module, as shown in **Table 4**.

**Table 4:** Ablation experiments.

Ablation Modules	OA(%)	AA(%)	mIoU(%)
Baseline	92.43	90.34	83.51
Baseline+PReLU	92.92	90.87	84.30
Baseline+Dice	93.38	91.67	85.52
Baseline+ECA	93.31	91.08	84.88
Baseline+CP Module	93.15	91.23	84.69
Baseline+Dice+ECA	93.70	91.70	85.52
Baseline+Dice+PReLU+ECA	93.52	**91.91**	85.75
Baseline+Dice+ECA+CP Module(afterECA)	93.72	91.82	85.80
Baseline+Dice+PReLU+ECA+CP Module(beforeECA)	93.37	91.09	84.79
Baseline+Dice+PReLU+ECA+CP Module(afterECA)	**93.74**	91.75	**85.99**

We tried different modules and different insertion sequences, and the main experimental results are as follows. Bold font indicates the highest performance.

In the upper half of **Table 4**, we mainly verified the impact of each module on the baseline. First, PReLU can better activate the nodes in our baseline network, with 0.49% and 0.79% improvement in OA and mIoU compared with ReLU. Secondly, both the dice loss function and the ECA module can improve the baseline network in all aspects. Dice loss function instead of cross entropy makes the baseline network achieve 0.95%, 1.33%, and 2.01% improvement in the three evaluation indexes. This is mainly because the calculation principle of the Dice loss function has a strong correlation with mIoU, which enables it to play the role of class balance. ECA makes the baseline network pay better attention to the key features through the channel attention mechanism, thus achieving 0.88%, 0.74%, and 1.37% improvement in the three evaluation indicators. Finally, the CP module enables the baseline network to achieve 0.72% and 1.18% improvement in OA and mIoU. This is mainly due to the additional performance improvement brought about by reclassification at difficult classification points.

In the lower part of **Table 4**, we mainly verified the role of different arrangements and combinations of modules in our method. First, the combination of the ECA module and Dice played the most important role in our method. It achieved 1.27% and2.01% improvement in OA and mIoU, respectively. Secondly, based on the ECA module and Dice, the addition of PReLU caused a decrease of 0.18% in OA and an increase of 0.23% in mIoU and obtained the highest AA. Thirdly, we placed the CP module that inputs the second-level resolution feature map before and after the ECA module to observe the change in its performance. Compared with the CP module not added, the CP module placed in front of the ECA module failed to achieve satisfactory results, while the CP module placed after the ECA module made our network achieve 0.22% and 0.24% improvement in OA and mIoU. It is worth mentioning that if too many feature maps with different levels of resolution are input into the CP module, the performance of the CP module

will decline. We also noticed that although PReLU caused a slight decrease in OA when combined with the ECA module and Dice, the addition of PReLU was still improved after the CP module was added or only the baseline network was used.

**Figure 6:**
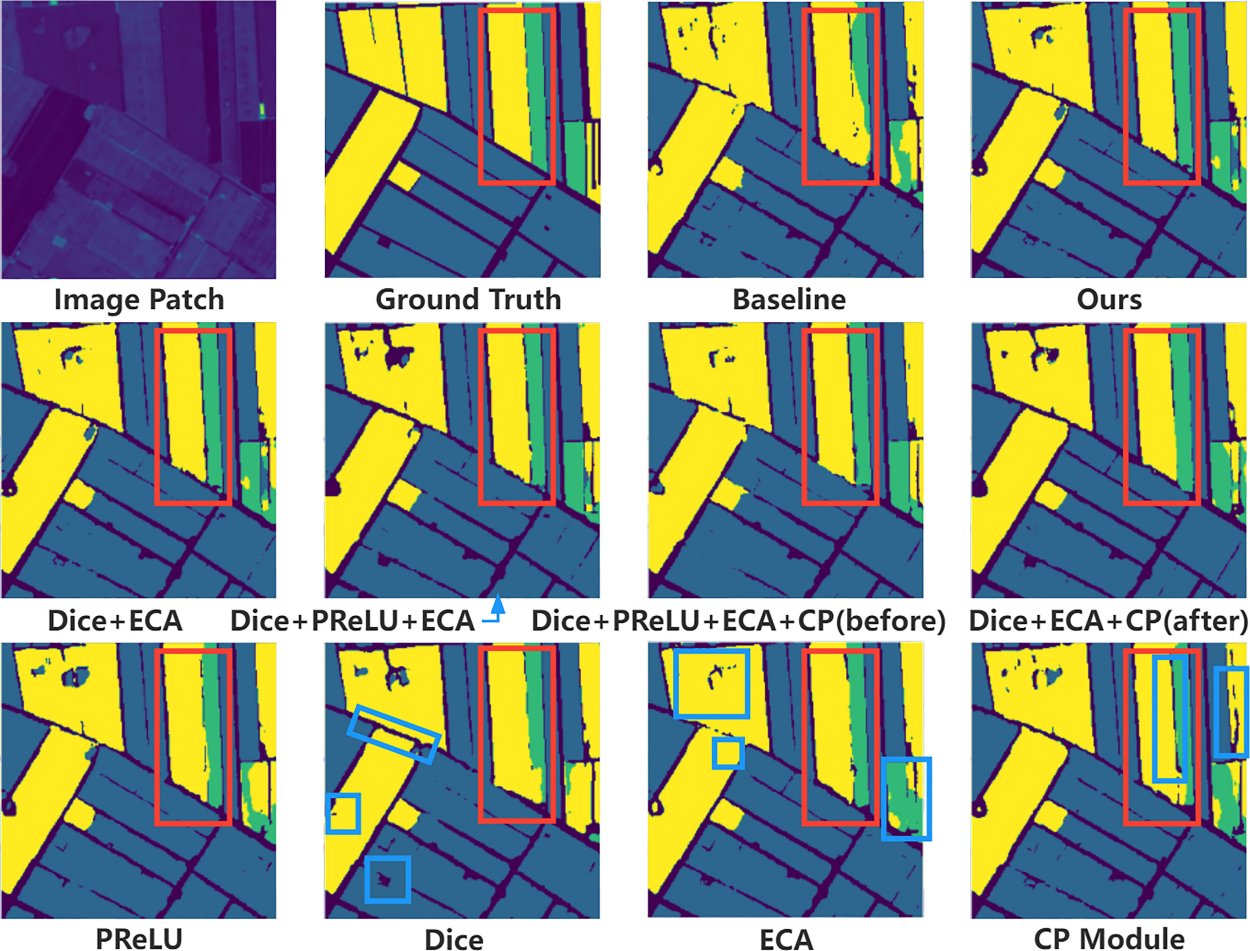
Visualization of the predicted results of ablation experiments. This part mainly analyzes the specific impact of adding and deleting different modules on the performance of the model. The second line of the image shows the common influence of different modules on the prediction results, and the third line shows the specific influence of each module on the prediction results. Among them, the red box shows the areas where our method is superior to other methods, and the blue box shows the areas where each module improves the baseline network.

The correct classification of the tiny parts of the image will not cause huge numerical changes in the evaluation indicators. Therefore, we analyze the specific impact of different modules in the model through visualization, and the visualization results are shown in **Figure 6**. First of all, through the visualization of the prediction results of each module, we can see that the Dice loss function has a good classification accuracy for the boundaries of some parcels, and can more accurately identify irregular shapes and gaps. The ECA module has a significant impact on the correctness of the classification of land parcel categories. It can be seen that the problem of the appearance of other types of prediction results in the same type of parcels has been suppressed. The CP module improves the details in many areas, mainly because its principle is aimed at points that are difficult to classify. It can be seen from the blue box on the far right of the visualization diagram of the CP module that it is the only model to improve the prediction accuracy of the wide parcel spacing area in the image. Compared with other models in the ablation experiment, our model has advantages in the accuracy of parcel boundary and classification of crop categories. The PReLU activation function is mainly a global promotion, so there are no specific areas circled with the blue box. In addition, in the visualization of the common influence of different modules, it can be seen that the performance is better than that of a single module.

For detailed experiments on the attention module and loss function used in the ablation experiments, see the next two subsections.

### 3.3 Loss function experiments

In the design of the loss function, we introduce the Dice into the loss function of the master branch of the model to alleviate the impact of class imbalance. We mixed Dice and cross-entropy with different weight ratios in our experiments to find the best combination for our dataset. The specific experiments are shown in **Table 2**.

The final experimental results show that using only Dice has the most obvious improvement in model performance. Due to the calculation principle of Dice, the improvement of the mIoU is higher than the improvement of the accuracy. Second, using a loss function that mixes cross-entropy and Dice at a ratio of 1:10 achieves the same improvement in OA, but decreases in mIoU.

### 3.4 Attention experiments

In the selection of attention modules, we consider channel attention, spatial attention, and channel & spatial attention. And we use SE Hu et al. (2020) and ECA two types of channel attention modules, CCA Huang et al. (2019) A Non-local self-attention module, as well as two types of channel & spatial attention modules, CBAM Woo et al. (2018) and Coordinate Attention (CA) Hou et al. (2021). Finally, we chose the best-performing ECA on our dataset as the attention used in our model. The specific experimental results are shown in **Table 5**.

**Table 5:** Insert different state-of-the-art attention modules into the backbone network for experiments.

Attention Module	OA(%)	AA(%)	mIoU(%)
Baseline Only	92.43	90.34	83.51
CBAM	92.79	90.71	84.24
SE	93.09	90.75	84.12
CA	93.11	90.75	84.21
CCA	93.27	90.67	84.27
ECA	**93.31**	**91.08**	**84.88**

Bold font indicates the highest performance.

It can be seen from the table that the ECA module has advantages over other attention modules for the baseline network. As channel attention, the ECA module performs much better than the SE module in our tasks. This is mainly because the ECA module avoids the reduction of feature dimension through the local cross-channel strategy. In addition, in the contrast experiments of the paper in which the ECA module is proposed, the promotion of the ECA module in the shallow network is significantly better than that in the deep network.

## 4 Discussion

Deep learning methods have shown significant advantages over traditional methods in various fields, such as semantic segmentation. However, deep learning is developing rapidly. It is a challenge to apply state-of-the-art technology to the subdivision field, especially to make certain adjustments to better solve the technical bottleneck in the subdivision field. An early study of deep learning applied to crop type mapping appeared in Zhong et al. (2019), which only used a simple fully-connected neural network structure. In our background investigation, there are a few studies were found that link crop type mapping to 2D semantic segmentation. In crop type mapping, the application of deep learning still has great research space and potential.

Most semantic segmentation networks are designed on standard computer vision benchmark datasets, typically large public datasets of typical color images. Typical color images are 3-band RGB images, while remote sensing images usually have more than three bands. In our experiments, we tried DeepLab v3+, and we adopted two schemes respectively. One is using RGB 3-band data for model training with pre-training. Another is using 4-band data for model training without pre-training. In contrast, the former scheme can achieve better performance. However, it has been verified in other models that do not require pre-training, and the addition of near-infrared light bands can significantly improve the performance of the model. Therefore, the DeepLab v3+ loses too many latent features due to this limitation. In our actual experiments, we also found that the backbone of DeepLab v3+ used, ResNet, is also very incompatible with the crop type mapping dataset, mainly because small datasets do not require too deep convolution layers to extract features.

In our work, we separately verify the effects of different architectures of CNNs on crop type mapping, and actively explore whether semantic segmentation modules, which have been proven effective in different domains, also have good performance in our network. In this paper, we identify the significant advantages of shallow convolutional neural networks on a small dataset to accomplish crop type mapping. CACPU-Net is influenced by many other network structures in the design process. For the combination of the attention module and CNN architecture, we refer to the multi-stage attention structure of MAResU-Net Li et al. (2022). Although MAResU-Net has not worked well in our dataset, we think this is more from the influence of the depth of the network. The design of the attention module is still worth learning. Among many attention modules, we finally chose ECA after experiments. We believe that the advantages of ECA in crop type mapping are mainly because the spectral features of agricultural remote sensing images can be well captured by this channel attention mechanism.

Our experiments show that CACPU-Net is more sensitive to the classification of parcel boundaries, which is also the advantage of using single temporal remote sensing images for crop type mapping. In addition, our dataset is relatively easy to produce, which can avoid errors caused by various operations such as image registration in the production of a multi-temporal dataset. Our research also has some defects. The main disadvantage is that our method is not compared with the multi-temporal method on the same dataset, which is mainly due to the large difference in the crop growth cycle between the public crop type mapping dataset and our dataset. Our next work plan is to expand our dataset, make a multi-temporal crop type mapping dataset for our experimental selection region, and complete the comparison with the multi-temporal crop type mapping method on this basis(In addition to the multi-temporal crop type mapping that has been proposed, considering that there are many well-performing 3D networks in medical image segmentation, such as nnU-Net Isensee et al. (2020) that outperforms in multiple different medical image segmentation datasets, we will try to implement in multi-temporal crop type mapping). In addition, inspired by Rundo et al. (2022), we plan to introduce a nested cross-validation scheme in future work to mitigate the negative impact of the lack of an independent external test dataset. Nested cross-validation is a model selection scheme, which can inhibit the overfitting of models. It applies to small datasets and is very suitable for our dataset. There are also some improvement schemes for nested cross-validation that we will consider together. Parvandeh et al. (2020) proposes consensus nested cross-validation, which can reduce the calculation cost of nested cross-validation. After the comparison with the multi-temporal crop type mapping method, our method can be widely used in all regions where crops are harvested in one season.

In general, our designed CACPU-Net can well meet the requirements of crop type mapping and achieve state-of-the-art performance on the dataset we made. The effect between different modules in CACPU-Net has improved the model to a certain extent. We have shown that the relationship between single-temporal remote sensing image features and crop refinement types is learnable.

## 5 Conclusion

A new convolutional neural network architecture with an attention mechanism (CACPU-Net) for crop type mapping is expected to become a general method for crop type mapping. Compared with the method for time series data, the method proposed in this paper has a lower amount of data and the difficulty of data collection and production in crop type mapping, which effectively reduces the number of model parameters. At the same time, our method achieves higher accuracy than other semantic segmentation methods. Our method improved the classification accuracy of parcel boundaries, which is mainly due to the Dice loss and CP module. The ECA module improved the sensitivity of the model to the crop type. Under the 10-fold cross-validation experiment, our model finally achieved 93.74% accuracy and 85.99% mIoU.

In future work, considering the difference in crop growth cycles in different climates, we plan to expand our dataset to a time series dataset and design the corresponding multi-temporal crop type mapping model. This allows us to assess the differences in the specific impact of time series data and single temporal data on crop type mapping and can be directly compared with other methods on public datasets, such as PASTIS Garnot and Landrieu (2021). In addition, we will pay more attention to the classification accuracy of parcel boundaries. Early crop type mapping research paid little attention to the parcel boundary Rußwurm and Körner (2018); Rustowicz et al. (2019). Garnot and Landrieu (2021) realized object-level parcel segmentation and improved the classification accuracy of the parcel boundary by introducing new labels and designing PaPs modules. We believe that introducing additional labels to limit the classification of the model on the parcel boundary (crop and non-crop parcels or parcel boundary labels, both of which can be generated through the original labels) can effectively improve the performance of the model, which is also the direction of our next work. We will also continue to design the loss function of the model through the research on the loss function in medical image segmentation (Ma et al., 2021; Yeung et al., 2022) to mitigate the impact of the imbalance problem of the dataset.

## Data availability statement

The original contributions presented in the study are included in the article/supplementary material. Further inquiries can be directed to the corresponding author.

## Author contributions

YB and WJ proposed linking 2D semantic segmentation to crop type mapping and the network architecture design. LL processed the original data and proposed the scheme of data clipping and mask. YB and LL performed the experiments and analyzed the data. YB wrote and revised the paper. LL and WJ provided valuable advice for the experiments and writing. All authors contributed to manuscript revision, read, and approved the submitted version.
